# Therapeutic experience with tramadol for opioid dependence in a patient with chronic low back pain: a case report

**DOI:** 10.1186/s40981-019-0289-z

**Published:** 2019-10-30

**Authors:** Marie Shigematsu-Locatelli, Takashi Kawano, Tsuyoshi Koyama, Hideki Iwata, Atsushi Nishigaki, Bun Aoyama, Hiroki Tateiwa, Noriko Kitaoka, Masataka Yokoyama

**Affiliations:** Department of Anesthesiology and Intensive Care Medicine, Kochi Medical School, Kohasu, Oko-cho, Nankoku, Kochi 783-8505 Japan

**Keywords:** Opioid dependence, Tramadol, Chronic pain

## Abstract

**Background:**

Long-term opioid treatment for chronic non-cancer pain has become controversial, given the increasing prevalence of opioid dependence. However, there is little information on therapeutic strategies for this condition in Japanese patients. Here, we present a case of successful management of iatrogenic opioid dependence with tramadol in a patient with chronic low back pain.

**Case presentation:**

A 68-year-old male suffering from intractable low back pain was referred to our pain clinic. He was previously treated in another hospital with transdermal fentanyl patches 6 mg/day and fentanyl sublingual tablets (100 μg as required) for this condition. On the basis of medical examination, including a review of the patient’s medical history, physical examination, X-ray, and his family statement, we diagnosed him with iatrogenic opioid dependence due to inadequate fentanyl use. Then, we developed a treatment plan consisting in fentanyl detoxification with a weak opioid, tramadol. At first, the use of fentanyl sublingual tablets was interrupted after obtaining informed consent. Then, we reduced the dose of transdermal fentanyl 1 mg *per* 4–5 days replacing with oral sustained-release tramadol. The patient developed mild to moderate withdrawal symptoms during this period, which could be effectively managed by supportive treatments. The hospital psychiatry liaison team continuously provided the patient and his wife with information, counseling, and education regarding the treatment of opioid dependence. Throughout the detoxification process, his reported pain did not exacerbate, even slightly improved over time. The final prescription was sustained-release tramadol 300 mg/day without fentanyl, and his activities of daily living drastically improved. However, unfortunately, he died due to an aortic dissection of stent-graft edge 65 days after surgery.

**Conclusions:**

Our case highlighted that sustained-release tramadol could be effectively applied as a detoxification agent for iatrogenic opioid dependence in patients with chronic non-cancer pain.

## Background

Opioid use disorders are well-known for leading to major health, social, and economic burden [1]. Specifically, iatrogenic opioid dependence or addiction is an emerging problem worldwide associated with increasing prescription rates. A recent meta-analysis shows an incidence of 4.7% within the patients that received opioid analgesic therapy [2]. In contrast, Japan has been considered to present one of the lowest incidences of inadequate opioid use due to two regulations, the narcotics control law and health care insurance system [3]. However, since 2010, opioid therapies including transdermal fentanyl patch were officially approved for chronic non-cancer pain. Therefore, although methamphetamine has been a more problematic substance than other drugs [4], there is ongoing concern regarding an increase in opioid use disorders. Based on such situation, the Japanese guideline for prescribing opioid analgesics for chronic non-cancer pain established the maximum daily dose as 120 mg morphine milligram equivalent (MME) and emphasized the importance of preventing the development of opioid use disorders [5]. Meanwhile, there is a lack of information regarding the treatment strategies of iatrogenic opioid dependence in Japan. Here, we present a successful case of iatrogenic opioid dependence treatment using tramadol in a patient with intractable chronic low back pain.

## Case presentation

**Fig. 1:**
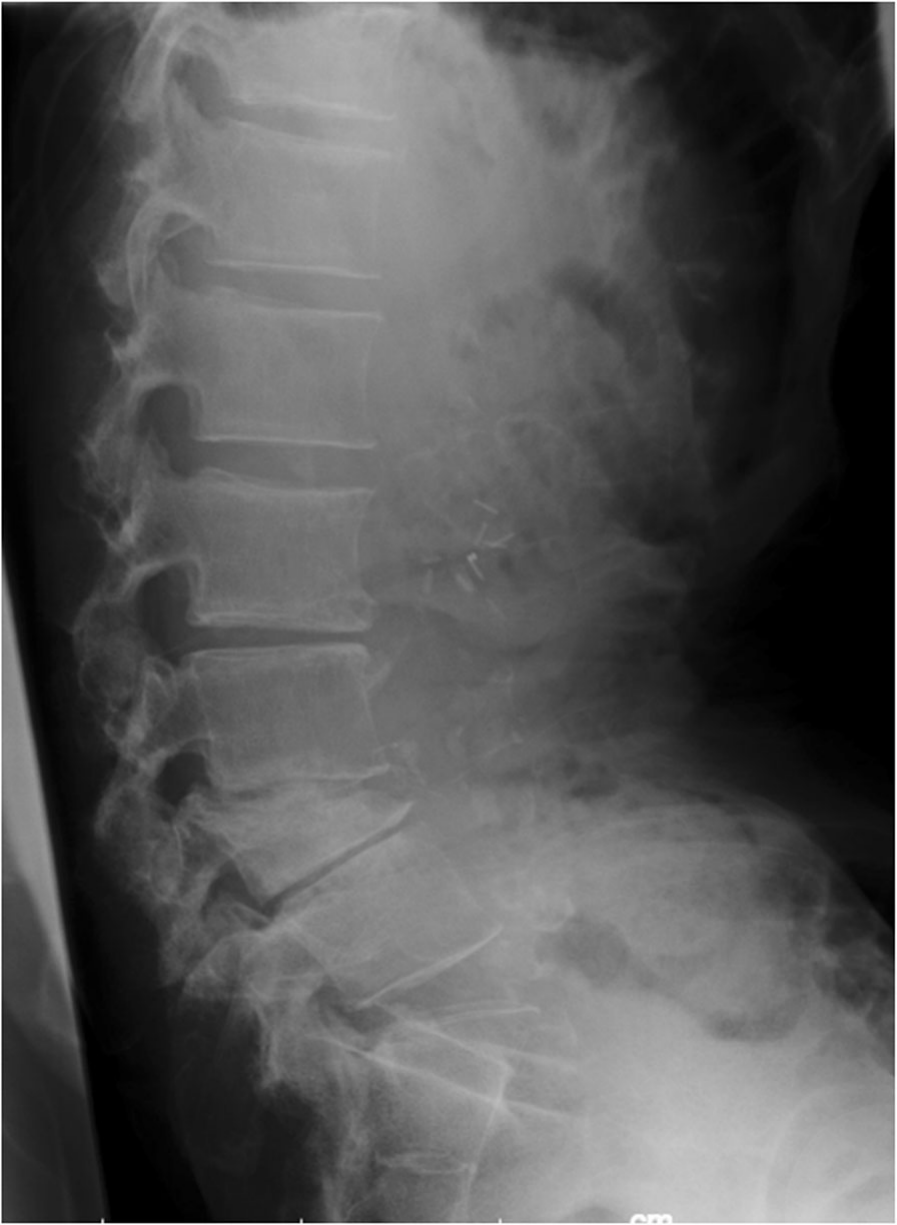
A radiograph of lateral lumbar spine. Compression fracture of L4 vertebral body is observed

A 68-year-old male suffering from intractable low back pain was referred to our pain clinic. He was previously treated in another hospital with transdermal fentanyl patches *Fentos Tape®* 6 mg/day and fentanyl sublingual tablets *Abstral®* 100 μg as required (usually 0–2 times/day) for this condition. He was originally admitted to our hospital for a stent-graft insertion surgery for thoracic aorta aneurysm. Although the patient’s postoperative pain was not predominant, his major complaint was regarding a worsening in continuous low back pain, leading to refuse rehabilitation. X-ray images showed an old L-4 lumbar compression fracture (Fig. 1), whereas we considered it may not sufficiently explain his severe pain. He also started to present behavioral problems confronting to the medical staff when requesting for more opioids. After interviewing his wife, we determined that the patient may be using the fentanyl sublingual tablets not for pain control but for anxiety reduction. The resident physician and his attending nurse witnessed and recorded short myoclonus-like convulsions during the nighttime.

We suspected opioid-related disorders and investigated the patient’s medical history for inadequate opioid use. His wife said that his personality used to be active, social, and industrious. However, 2 years before, he was diagnosed as terminal stage of cholangiocarcinoma by his family practitioner. This may be a misunderstanding, because his medical examinations including abdominal CT scans performed in our hospital revealed no sign of cancer. Erroneously believing that he had cancer, the patient became depressed, refusing further examinations, and his low back pain worsened. He also misunderstood that his low back pain was related to cancer metastasis. First-line analgesics, including acetaminophen, nonsteroidal anti-inflammatory drugs, and duloxetine, were prescribed, but none yielded satisfactory results. The patient only requested opioid prescription of transdermal fentanyl for his lumbar pain. His medical record showed that he had visited three different hospitals for his intractable pain symptom. During this period, the dose of transdermal fentanyl was gradually increased, as well as prescribed fentanyl sublingual tablet on an as-needed basis.

**Fig. 2:**
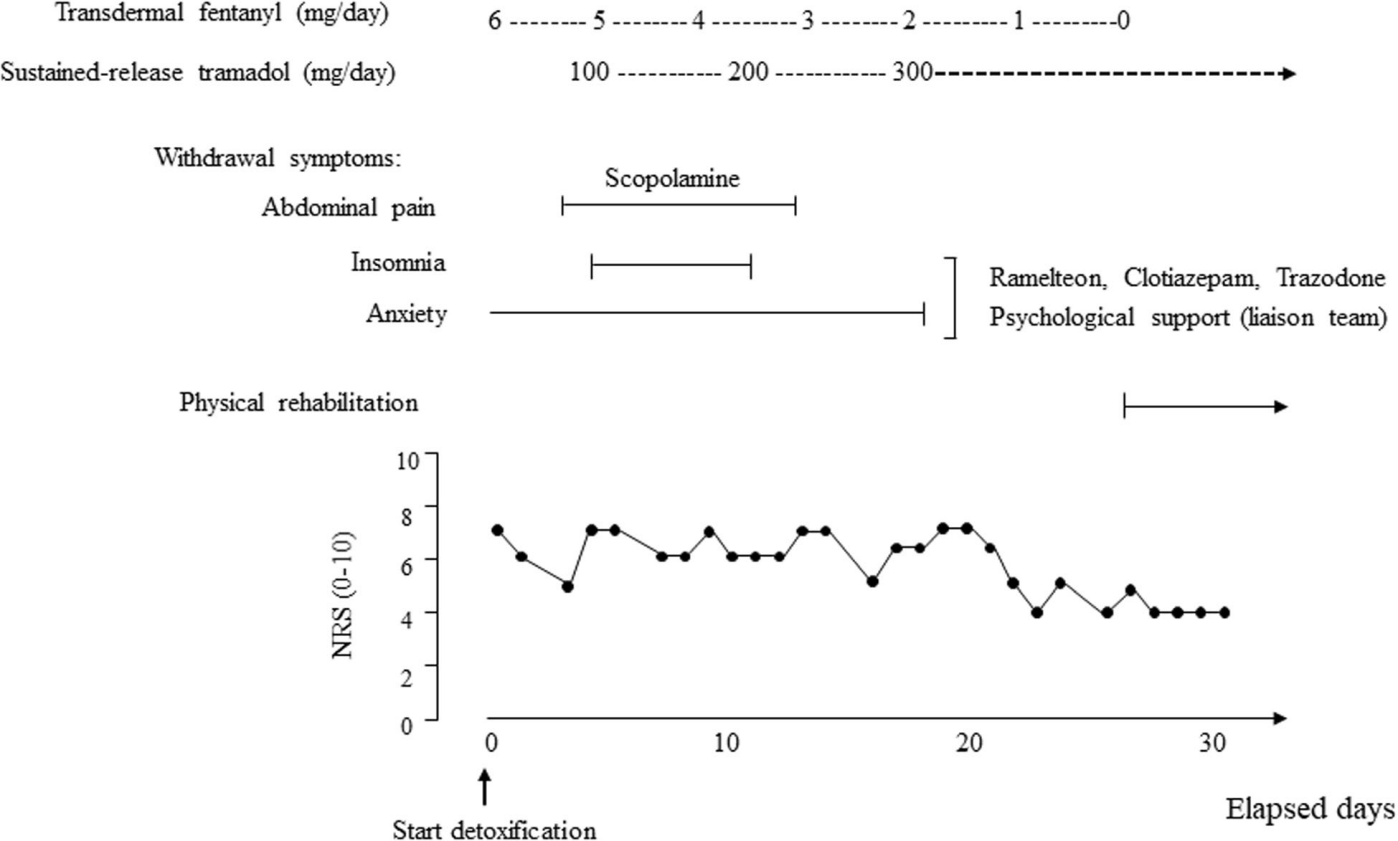
Time course of fentanyl detoxification with sustained-release tramadol. Pain intensity was assessed using the NRS (Numerical Rating Scale)

At the beginning, in order to rule out the possibility of opioid tolerance and pseudoaddiction, we switched opioids from fentanyl to equivalent dose of oral morphine (controlled-release formulations, *P-guard®*). However, no significant changes were observed in the pain symptom, as well as in the frequency of fentanyl sublingual tablet requirement. After that, the oral morphine was reverted to the transdermal fentanyl. Then, according to the International Statistical Classification of Diseases and Related Health Problems (ICD)-10 [6], we diagnosed the patient with opioid-related dependence (Additional file 1). In consequence, we developed a treatment plan that focused on fentanyl detoxification and replacement therapy with a weak opioid, tramadol. Before beginning treatment, we carefully explained to the patient and his wife about inappropriate fentanyl use and suspected opioid dependence. At first, in collaboration with the palliative care team, we informed them the importance of interrupting the use of fentanyl sublingual tablets and obtained their consent. Next, we reduced the dose of transdermal fentanyl 1 mg *per* 4–5 days replacing with oral tramadol (*Onetram®*, sustained-release tablet) (Fig. 2). During this treatment period, the *patient* developed mild to moderate *withdrawal symptoms*, such as abdominal pain with diarrhea, insomnia, and anxiety. These symptoms could be effectively managed by supportive treatment, i.e., abdominal pain was treated by scopolamine butyl bromide, and the insomnia and anxiety were controlled with ramelteon 8 mg, clotiazepam 5 mg, and trazodone hydrochloride 25 mg. The patient did not complain about pain exacerbation throughout the treatment (Fig. 2), and no additional analgesic was required. The hospital psychiatry liaison team continued to provide the patient and his wife with information, counseling, and education regarding the treatment of opioid dependence. After the completion of our treatment program, his activities of daily living drastically improved, and the patient could start rehabilitation. The final prescription was tramadol 300 mg/day without fentanyl, which continued to be prescribed for his lower back pain. However, unfortunately, he died due to an aortic dissection of stent-graft edge 65 days after surgery.

## Discussion

We described a case of iatrogenic opioid dependence induced by inadequate fentanyl use for chronic non-cancer pain. The patient was successfully detoxified by sustained-released tramadol without severe withdrawal symptoms.

Iatrogenic opioid dependence is a serious adverse effect associated with long-term opioid therapy for chronic non-cancer pain [1, 7]. In order to prevent its development, it is critically important for the physician to prescribe opioids appropriately. Specifically, since high-dose opioid prescription is one of the major risk factors, the Japanese clinical practice guideline recommends the maximum daily opioid dose as 120 mg of MME [5]. In addition, sublingual formulation of fentanyl can be used to achieve a rapid onset of action, which may enhance abuse potential [8]. Accordingly, it is only indicated for breakthrough pain in opioid-treated cancer patients. In our case, however, the patient treated his chronic low back pain with transdermal fentanyl 6 mg (180 mg of MME) in combination with sublingual fentanyl tablets. This inappropriate opioid use may be a consequence of complicated circumstances, such as patient misinterpretation, patient-physician miscommunication, inadequate physician attitudes towards opioids, and lack of drug monitoring systems.

Inadequate pain management led to drug-seeking behaviors that resemble those seen with opioid addiction. This phenomenon is referred to as pseudoaddiction [9, 10]. In clinical settings, pseudoaddiction can be distinguished from opioid dependence in that the behaviors disappear after appropriate pain treatment. On the other hand, opioid tolerance develops with repeated opioid exposure, resulting in a decrease in the analgesic effect [10, 11]. The development of opioid tolerance has been reported to lead to an increase in opioid consumption and the risk of addiction. Opioid switching or rotation is the process of substituting one opioid for another for improvement of opioid responsiveness [11]. In our case, despite an opioid switching from transdermal fentanyl to equi-analgesic dose of oral morphine, no significant pain relief was observed. Furthermore, his pain intensity did not exacerbate during fentanyl detoxification, even presented a slight improvement over time (Fig. 2), indicating that the occurrence of pseudoaddiction could be excluded.

A crucial first step in the treatment of opioid dependency is detoxification [12, 13]. Opioid detoxification is the supervised withdrawal from causative opioid to minimize withdrawal symptoms. Currently, two types of Food and Drug Administration (FDA)-approved medications, oral methadone and sublingual buprenorphine, are widely used as the standard agents for medical opioid detoxification [12, 13]. Methadone is a full opioid agonist with long half-life and high affinity at the μ-opioid receptor, which can ameliorate the euphoria effects and withdrawal symptoms [14]. Buprenorphine is a partial opioid agonist, which makes it an advantageous alternative to methadone due to its relatively safe profile [15]. Evidence shows that both medications help patients to reach a stabilized physical condition, which then allows them to successfully go through counseling and rehabilitation programs [12–15]. However, they are not currently prescribed in Japan, i.e., oral methadone is only indicated for the treatment of cancer pain, and sublingual formulation of buprenorphine has not been available yet.

Tramadol is a centrally acting analgesic for treatment of moderate to severe pain in both cancer and non-cancer patients [16]. Since it binds to μ-opioid receptors, tramadol could relieve opioid withdrawal symptoms [17]. In fact, a previous clinical trial showed that oral treatment of immediate-release tramadol (200 or 400 mg) reduces opioid withdrawal in morphine-maintained adults [18]. Another report further demonstrated that the extended-release tramadol of therapeutic dose (200 mg daily) was more efficacious than placebo and supra-therapeutic dose (600 mg daily) in treating withdrawal in opioid dependence patients [19]. In contrast, a recent randomized controlled trial showed that higher doses of extended-release tramadol (up to 600 mg daily) suppressed opioid withdrawal symptom comparably to buprenorphine [20]. Therefore, relatively small dose regimen (up to 300 mg daily) may explain why some withdrawal symptoms were developed in our case.

On the other hand, the affinity of tramadol for the μ-opioid receptor is very low, i.e., 6000-fold less than that of morphine [16]. It may be associated with less incidence of side effects, including lower rates of respiratory depression and reduced abuse liability, compared to other μ-opioid analgesics. Specifically, compared with immediate-release formulations, sustained-release tramadol could provide a more controlled, safe, and prolonged analgesic effect, minimizing adverse effects associated with uncontrolled drugs peak plasma levels [21]. Furthermore, tramadol inhibits the neuronal re-uptake of noradrenaline and serotonin, which contributes to better analgesic effects for chronic pain via activation of descending inhibitory pathways [16, 21]. These pharmacological profiles, along with our case, suggest that the medication with sustained-release tramadol could be an effective alternative approach for supervised opioid detoxification in patients with chronic non-cancer pain. Nevertheless, several important questions remain such as long-term effectiveness and optimal dose regimen. In addition, a Cochrane database systemic review published in 2018 reported that there were insufficient data to determine whether tramadol differs from methadone in alleviating withdrawal symptoms [17]. Therefore, further clinical studies regarding our hypothesis are necessary and warranted.

## Conclusion

Although detoxification is one essential component of an effective treatment for opioid dependence, none of the FDA-approved medications are currently available in Japan. Our case highlighted that sustained-release tramadol could be a safe alternative detoxification agent for opioid dependence in patients with chronic non-cancer pain.

## Supplementary information

**Additional file 1.** Diagnosis of opioid-related dependence. (DOCX 13 kb)

## Data Availability

The data are not available for public access because of patient privacy concerns, but are available from the corresponding author on reasonable request.

## Abbreviations

FDA: Food and Drug Administration
ICD: International Statistical Classification of Diseases and Related Health Problems
MME: Morphine milligram equivalent
