# Supplementary material for: Therapeutic experience with tramadol for opioid dependence in a patient with chronic low back pain: a case report
Source: JA Clin Rep. 2019 Oct 30;5:68. doi: 10.1186/s40981-019-0289-z (PMC6967209; doi:10.1186/s40981-019-0289-z)
Supplement: Supplementary file 1 — Additional file 1. Diagnosis of opioid-related dependence. (DOCX 13 kb) [file 40981_2019_289_MOESM1_ESM.docx]

**Additional file 1**

In the International Statistical Classification of Diseases and Related Health Problems (ICD)-10, a definite diagnosis of dependence should usually be made only if three or more of the following have been present together at some time during the previous year:

(a) A strong desire or sense of compulsion to take the substance;

(b) Difficulties in controlling substance-taking behavior in terms of its onset, termination, or levels of use;

(c) A physiological withdrawal state when substance use has ceased or have been reduced, as evidenced by the characteristic withdrawal syndrome for the substance; or use of the same (or closely related) substance with the intention of relieving or avoiding withdrawal symptoms;

(d) Evidence of tolerance, such that increased doses of the psychoactive substance are required in order to achieve effects originally produced by lower doses (clear examples of this are found in alcohol- and opiate-dependent individuals who may take daily doses sufficient to incapacitate or kill nontolerant users);

(e) Progressive neglect of alternative pleasures or interests because of psychoactive substance use, increased amount of time necessary to obtain or take the substance or to recover from its effects;

(f) Persisting with substance use despite clear evidence of overtly harmful consequences, such as harm to the liver through excessive drinking, depressive mood states consequent to periods of heavy substance use, or drug-related impairment of cognitive functioning; efforts should be made to determine that the user was actually, or could be expected to be, aware of the nature and extent of the harm.

Our patient had all the symptoms except (c) and (d) when he was referred to our pain clinic. Thus, we diagnosed him as opioid dependence syndrome.
